# Replication of Established Common Genetic Variants for Adult BMI and Childhood Obesity in Greek Adolescents: The TEENAGE Study

**DOI:** 10.1111/ahg.12012

**Published:** 2013-01-24

**Authors:** Ioanna Ntalla, Kalliope Panoutsopoulou, Panagiota Vlachou, Lorraine Southam, Nigel William Rayner, Eleftheria Zeggini, George V Dedoussis

**Affiliations:** 1 Harokopio University of Athens, Department of Nutrition and DieteticsAthens, Greece; 2 Wellcome Trust Sanger Institute, The Morgan Building, Wellcome Trust Genome CampusHinxton, Cambridge, United Kingdom; 3 Wellcome Trust Centre for Human Genetics, University of OxfordOxford, United Kingdom; 4 Oxford Centre for Diabetes Endocrinology and Metabolism, Churchill HospitalHeadington, Oxford, United Kingdom

**Keywords:** Obesity, BMI, common genetic variants, adolescents

## Abstract

Multiple genetic loci have been associated with body mass index (BMI) and obesity. The aim of this study was to investigate the effects of established adult BMI and childhood obesity loci in a Greek adolescent cohort. For this purpose, 34 variants were selected for investigation in 707 (55.9% females) adolescents of Greek origin aged 13.42 ± 0.88 years. Cumulative effects of variants were assessed by calculating a genetic risk score (GRS-34) for each subject. Variants at the *FTO, TMEM18, FAIM2*, *RBJ, ZNF608* and *QPCTL* loci yielded nominal evidence for association with BMI and/or overweight risk (*p* < 0.05). Variants at *TFAP2B* and *NEGR1* loci showed nominal association (*p* < 0.05) with BMI and/or overweight risk in males and females respectively. Even though we did not detect any genome-wide significant associations, 27 out of 34 variants yielded directionally consistent effects with those reported by large-scale meta-analyses (binomial sign *p* = 0.0008). The GRS-34 was associated with both BMI (beta = 0.17 kg/m^2^/allele; *p* < 0.001) and overweight risk (OR = 1.09/allele; 95% CI: 1.04–1.16; *p* = 0.001). In conclusion, we replicate associations of established BMI and childhood obesity variants in a Greek adolescent cohort and confirm directionally consistent effects for most of them.

## Introduction

Prevalence of overweight and obesity in adolescents has increased over the last decades, and the observed rates in Greece are higher or comparable to those reported for other European countries (Lobstein & Frelut, 2003; Tzotzas et al., 2008). Although the increased obesity levels have been attributed to environmental changes, a strong genetic component has also been shown to contribute. Genome-wide association studies (GWAS) have been successful in identifying multiple genetic loci associated with BMI and/or obesity (Frayling et al., 2007; Scuteri et al., 2007; Loos et al., 2008; Thorleifsson et al., 2009; Willer et al., 2009; Speliotes et al., 2010; Bradfield et al., 2012).

Large GWAS meta-analyses, from the Genetic Investigation of Anthropometric Traits (GIANT) (Speliotes et al., 2010) and Early Growth Genetics (EGG) consortia (Bradfield et al., 2012), have identified multiple BMI-associated loci. The GIANT meta-analysis (Speliotes et al., 2010) of 249,796 individuals confirmed 14 established obesity susceptibility loci and identified 18 new loci associated with adult BMI. Twenty-three of the 32 variants also showed directionally consistent effects on children's BMI. The EGG meta-analysis replicated associations for seven established adult BMI variants and identified two novel loci robustly associated with increased childhood obesity risk (Bradfield et al., 2012).

In this study, we examined the effects of established adult BMI and childhood obesity associated loci reported by GIANT (Speliotes et al., 2010) and EGG (Bradfield et al., 2012) meta-analyses respectively on BMI and overweight risk in Greek adolescents. In total 34 single nucleotide polymorphisms (SNPs) were selected for investigation; 32 were based on the GIANT loci (*FTO, MEM18, MC4R, GNPDA2, BDNF, NEGR1, SH2B1, ETV5, MTCH2, KCTD15, SEC16B, TFAP2B, FAIM2, NRXN3, RBJ, GPRC5BC, MAP2K5, QPCTL, TNNI3K, SLC39A8, FLJ35779, LRRN6C, TMEM160, FANCL, CADM2, PRKD1, LRP1B, PTBP2, MTIF3, ZNF608, RPL27A, NUDT3*) (Speliotes et al., 2010); and two were based on the EGG loci (*OLFM4, HOXB5*) (Bradfield et al., 2012).

## Materials and Methods

Our sample comprised 707 (55.9% females) adolescents of Greek origin aged 13.42 ± 0.88 years. Participants were drawn from the TEENAGE (TEENs of Attica: Genes and Environment) study. A random sample of 857 adolescent students (Table S1) attending public secondary schools located in the wider Athens area of Attica in Greece were recruited in the study from 2008 to 2010. Details of recruitment and data collection have been described elsewhere (Ntalla et al*., in press*). Prior to recruitment all study participants gave their verbal assent along with their parents’/guardians’ written consent forms. The study was approved by the Institutional Review Board of Harokopio University and the Greek Ministry of Education, Lifelong Learning and Religious Affairs.

DNA samples were genotyped using Illumina HumanOmniExpress BeadChips (Illumina, San Diego, CA, USA) at the Wellcome Trust Sanger Institute, Hinxton, UK. Genotype calling algorithm used was Illuminus (Teo et al., 2007). Sample exclusion criteria included: (i) sample call rate < 95%; (ii) samples with sex discrepancies; (iii) samples with genome-wide heterozygosity of <32% or >35%; (iv) duplicated/related samples identified by calculating the genome-wide pair-wise identity by descent (IBD) for each sample using PLINK (Purcell et al., 2007); from each pair with a π∧ > 0.2 the sample with the lower call rate was excluded; (v) samples with evidence of non-European descent as assessed by performing multidimensional scaling (MDS) in PLINK (Purcell et al., 2007) by combining the TEENAGE dataset with 1184 individuals from the HapMap phase III populations. SNP exclusion criteria included: (i) Hardy Weinberg Equilibrium (HWE) exact *p* < 0.0001, (ii) MAF < 1%, (iii) call rate < 95% (for SNPs with MAF ≥ 5%) or call rate < 99% for SNPs with MAF < 5%. Genotypes were imputed using the directly typed data and phased HapMap II genotype data from the 60 CEU HapMap founders using the program IMPUTE (Marchini & Howie, 2010).

Body weight (kg) was measured to the nearest 0.1 kg with the subjects barefoot and dressed in light clothing by the use of a weighing scale (Seca Alpha, Hamburg, Germany). Height was measured to the nearest 0.1 cm using a portable stadiometer with participants being barefoot with their shoulders in a relaxed position, their arms hanging freely and their head in a normal position, with the eyes looking straight ahead. BMI was calculated as weight (kg)/height squared (m^2^). Subjects were classified as normal weight, overweight and obese according to the age- and sex-specific criteria adopted by the International Obesity Task Force (IOTF) (Cole et al., 2000).

Before testing for associations, BMI was log-transformed to achieve normal distribution. The association of each variant with BMI was tested using linear regression. Overweight and obese subjects were classified into one category, and overweight risk was tested using logistic regression. Obesity risk was also tested with the use of logistic regression. All analyses were adjusted for age and sex and were carried out assuming an additive genetic model in SNPTEST (Marchini et al., 2007). In order to investigate whether some of the selected variants have sex-specific effects, stratification analyses by sex were also performed. In order to investigate the cumulative effects of variants, a genetic risk score, comprising all selected variants for investigation (GRS-34) was calculated for each subject by adding the effect alleles of the SNPs. For imputed variants, we used best-guess genotypes to calculate the score, using a genotype probability threshold of 0.8. Given that it has been proposed that allele weighting may have a limited effect (Janssens et al., 2007), we did not weight risk alleles for their individual effect sizes. Linear and logistic regression models were performed in STATA-11 (StataCorp, College Station, TX) to investigate the effects of the score on BMI and overweight risk respectively. Quanto v1.2.4 (http://hydra.usc.edu/gxe/) was used for power calculations. A two-sided *p* ≤ 0.05 was used as the threshold for nominal significance, and *p* ≤ 5 × 10^−8^ was used as the threshold for genome-wide significance.

## Results

Three variants corresponding to previously identified GIANT loci (*FTO, TMEM18, and FAIM2*) yielded nominal evidence for association with both BMI (beta ± SE: 0.57 ± 0.19, *p* = 0.001; beta ± SE: 0.46 ± 0.24, *p* = 0.031; and beta ± SE: 0.49 ± 0.20, *p* = 0.015 respectively) and overweight risk (OR = 1.33, 95% CI: 1.06–1.67, *p* = 0.019; OR = 1.46, 95% CI: 1.08–1.97, *p* = 0.011; and OR = 1.29, 95% CI: 1.03–1.63, *p* = 0.025 respectively) in TEENAGE (Table 1). Variation at the *QPCTL* and *ZNF608* loci yielded nominal evidence for association with BMI (beta ± SE: 0.54 ± 0.25, *p* = 0.026 and beta ± SE: 0.38 ± 0.25, *p* = 0.047 respectively), while the index variant at the *RBJ* locus was associated with overweight risk (OR = 1.32, 95% CI: 1.05–1.66, *p* = 0.017). The strongest association was observed at the *FTO* locus with BMI, which also accounted for the largest proportion of variation (0.96%) (Table 1). Obesity risk was nominally associated with variation at the *FAIM2* locus (OR = 1.58, 95% CI: 1.05–2.40, *p* = 0.025), and with a variant at the *BDNF* locus (OR = 1.72, 95% CI: 1.00–2.95, *p* = 0.028) (Table S2). Despite the lack of statistically significant evidence for association for the majority of variants, overall 27 of the 34 GIANT and EGG loci yielded directionally consistent effects in TEENAGE (binomial sign test *p* = 0.0008) (Table 1).

**Table 1:** TEENAGE association summary statistics for BMI and childhood obesity associated loci

Consortial association summary statistics (GIANT and EGG)										TEENAGE association summary statistics							
		
											BMI^1^				Overweight risk^3^		
												
				Alleles													
																
SNP	Nearest gene	Chr	Position (bp)	Effect	Other	EAF	beta (or OR)	SE (or 95% CI)	*p*	EAF	beta^2^	SE^2^	Explained variation (%)	*p*	OR	95% CI	*P*
*GIANT* (Speliotes et al., 2010)^4^																	
**rs1558902**	***FTO***	**16**	**52361075**	**A**	**T**	**0.42**	**0.39**	**0.02**	**4.8E-120**	**0.48**	**0.57**	**0.19**	**0.96**	**0.001**	**1.33**	**1.06–1.67**	**0.019**
**rs2867125**	***TMEM18***	**2**	**612827**	**C**	**T**	**0.83**	**0.31**	**0.03**	**2.77E-49**	**0.80**	**0.46**	**0.24**	**0.95**	**0.031**	**1.46**	**1.08–1.97**	**0.011**
rs571312	*MC4R*	18	55990749	A	C	0.24	0.23	0.03	6.43E-42	0.26	0.04	0.22	0.93	0.941	1.00	0.77–1.30	0.938
rs10938397	*GNPDA2*	4	44877284	G	A	0.43	0.18	0.02	3.78E-31	0.42	0.11	0.20	0.65	0.490	0.99	0.79–1.25	0.855
rs10767664	*BDNF*	11	27682562	A	T	0.78	0.19	0.03	4.69E-26	0.75	0.32	0.23	0.64	0.188	1.16	0.89–1.51	0.229
rs2815752	*NEGR1*	1	72585028	A	G	0.61	0.13	0.02	1.61E-22	0.72	0.04	0.22	0.30	0.913	0.94	0.73–1.21	0.698
rs7359397	*SH2B1*	16	28793160	T	C	0.4	0.15	0.02	1.88E-20	0.29	-0.08	0.22	0.38	0.867	0.98	0.76–1.26	0.865
rs9816226	*ETV5*	3	187317193	T	A	0.82	0.14	0.03	1.69E-18	0.82	0.11	0.26	0.30	0.566	1.03	0.77–1.39	0.838
rs3817334	*MTCH2*	11	47607569	T	C	0.41	0.06	0.02	1.59E-12	0.41	0.02	0.20	0.33	0.720	0.81	0.64–1.02	0.083
rs29941	*KCTD15*	19	39001372	G	A	0.67	0.06	0.02	3.01E-09	0.67	0.05	0.21	0.35	0.840	1.19	0.94–1.52	0.158
rs543874	*SEC16B*	1	176156103	G	A	0.19	0.22	0.03	3.56E-23	0.13	0.16	0.29	0.85	0.449	1.00	0.71–1.40	0.954
rs987237	*TFAP2B*	6	50911009	G	A	0.18	0.13	0.03	2.9E-20	0.18	0.48	0.25	0.67	0.082	1.25	0.94–1.66	0.177
**rs7138803**	***FAIM2***	**12**	**48533735**	**A**	**G**	**0.38**	**0.12**	**0.02**	**1.82E-17**	**0.37**	**0.49**	**0.20**	**0.91**	**0.015**	**1.29**	**1.03–1.63**	**0.025**
rs10150332	*NRXN3*	14	79006717	C	T	0.21	0.13	0.03	2.75E-11	0.18	0.04	0.25	0.30	0.948	1.14	0.85–1.53	0.314
**rs713586**	***RBJ***	**2**	**25011512**	**C**	**T**	**0.47**	**0.14**	**0.02**	**6.17E-22**	**0.44**	0.26	0.19	0.41	0.187	**1.32**	**1.05–1.66**	**0.017**
rs12444979	*GPRC5BC*	16	19841101	C	T	0.87	0.17	0.03	2.91E-21	0.88	0.25	0.29	0.42	0.410	0.83	0.58–1.18	0.316
rs2241423	*MAP2K5*	15	65873892	G	A	0.78	0.13	0.02	1.19E-18	0.76	0.06	0.23	0.30	0.739	0.91	0.69–1.19	0.490
**rs2287019**	***QPCTL***	**19**	**50894012**	**C**	**T**	**0.8**	**0.15**	**0.03**	**1.88E-16**	**0.83**	**0.54**	**0.25**	**0.84**	**0.026**	0.82	0.60–1.11	0.187
rs1514175	*TNNI3K*	1	74764232	A	G	0.43	0.07	0.02	8.16E-14	0.40	0.02	0.19	0.41	0.740	0.97	0.77–1.22	0.831
rs13107325	*SLC39A8*	4	103407732	T	C	0.07	0.19	0.04	1.5E-13	0.10	0.51	0.32	0.40	0.084	1.33	0.93–1.90	0.122
rs2112347	*FLJ35779*	5	75050998	T	G	0.63	0.1	0.02	2.17E-13	0.60	0.04	0.19	0.36	0.704	1.01	0.80–1.27	0.898
rs10968576	*LRRN6C*	9	28404339	G	A	0.31	0.11	0.02	2.65E-13	0.22	-0.27	0.23	0.63	0.281	0.95	0.72–1.25	0.780
rs3810291	*TMEM160*	19	52260843	A	G	0.67	0.09	0.02	1.64E-12	0.68	-0.09	0.21	0.38	0.753	0.91	0.72–1.16	0.420
rs887912	*FANCL*	2	59156381	T	C	0.29	0.1	0.02	1.79E-12	0.28	-0.15	0.21	0.36	0.550	0.97	0.75–1.24	0.728
rs13078807	*CADM2*	3	85966840	G	A	0.2	0.1	0.02	3.94E-11	0.22	0.25	0.23	0.84	0.280	1.11	0.84–1.45	0.551
rs11847697	*PRKD1*	14	29584863	T	C	0.04	0.17	0.05	5.76E-11	0.07	0.51	0.39	0.71	0.229	1.00	0.63–1.58	0.942
rs2890652	*LRP1B*	2	142676401	C	T	0.18	0.09	0.03	1.35E-10	0.16	0.12	0.27	0.40	0.659	1.05	0.77–1.43	0.688
rs1555543	*PTBP2*	1	96717385	C	A	0.59	0.06	0.02	3.68E-10	0.50	0.20	0.19	0.50	0.154	1.15	0.92–1.44	0.220
rs4771122	*MTIF3*	13	26918180	G	A	0.24	0.09	0.03	9.48E-10	0.21	-0.11	0.25	0.35	0.623	0.94	0.71–1.25	0.679
**rs4836133**	***ZNF608***	**5**	**124360002**	**A**	**C**	**0.48**	**0.07**	**0.02**	**1.97E-09**	**0.53**	**0.38**	**0.20**	**0.49**	**0.047**	1.15	0.91–1.44	0.213
rs4929949	*RPL27A*	11	8561169	C	T	0.52	0.06	0.02	2.8E-09	0.37	-0.27	0.21	0.82	0.245	0.97	0.76–1.22	0.783
rs206936	*NUDT3*	6	34410847	G	A	0.21	0.06	0.02	3.02E-08	0.24	-0.02	0.23	0.32	0.928	0.86	0.66–1.13	0.298
*EGG* (Bradfield et al., 2012)^5^																	
rs9568856	*OLFM4*	13	52962982	A	G	0.16	1.22	1.14–1.29	1.82E-09	0.13	0.51	0.29	0.66	0.070	1.12	0.80–1.57	0.421
rs9299	*HOXB5*	17	44024429	T	C	0.65	1.14	1.09–1.20	3.54E-09	0.66	0.16	0.20	0.74	0.363	1.09	0.85–1.38	0.539

Chr = chromosome, bp = base pairs, EAF = effect allele frequency, SE = standard error, OR = odds ratio, CI = confidence interval.

Results were obtained using linear regression and logistic regression analysis assuming an additive effect while controlling for age and sex. Allelic test *p*, beta and SE, OR and 95% CIs are shown for each single SNP. Effect sizes (beta) and ORs are reported for the effect allele. Bold high-lighted loci yielded at least nominal evidence for association with BMI and/or overweight risk.

1 “BMI” refers to the linear regression analysis of each variant with BMI.

2 Effect sizes (beta) and SE are given for untransformed BMI (kg/m^2^).

3 “Overweight risk” refers to the binary trait analysis: normal weight subjects vs. overweight subjects (obese subjects were also classified as overweight).

4 Effect sizes in kg/m^2^ are referring to Stage 2 findings only; *p* are referring to Stage 1 and Stage 2 combined findings.

5 OR, CI and *p* are referring to the overall EGG consortium meta-analysis findings; EAF is referring to the discovery stage findings.

In the sex-stratified analysis, BMI and obesity were also nominally associated with a variant at the *TFAP2B* locus (beta ± SE: 1.22 ± 0.39, *p* = 0.002; OR = 1.96, 95% CI: 1.08–3.55, *p* = 0.026) (Table S3a) in males. In females, a variant at the *NEGR1* locus also yielded nominal evidence of association with risk of obesity (OR = 3.15, 95% CI: 1.10–9.02, *p* = 0.018). Variants at *MTCH2*, *LRRN6C* and *TMEM160* were also nominally associated with overweight or obesity risk. However, the direction of effects observed in our study for these variants was inconsistent with those reported from the GIANT consortium (Speliotes et al., 2010) (Table S3b).

**Figure 1:**
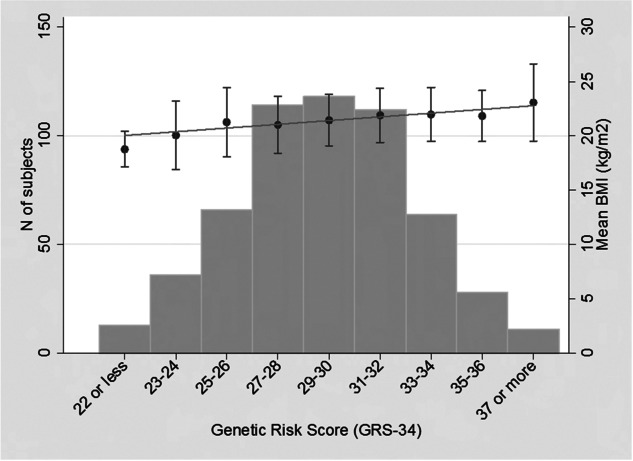
Distribution of the genetic risk score and cumulative effects of the risk alleles from 32 GIANT BMI and 2 EGG childhood obesity SNPs. Mean (±SE) values for BMI (kg/m^2^) are also presented.

The GRS-34, which was constructed to investigate the cumulative effects of the 34 variants under study, was significantly associated with BMI (beta = 0.17 kg/m^2^/allele; *p* < 0.001) and explained 3.2% of BMI variation. The difference in mean BMI between subjects with the higher GRS-34 (≥ 37 effect alleles) (2.0% of subjects) and those with the lower GRS-34 (≤ 22 effect alleles) (2.3% of subjects) was 4.3 kg/m^2^ (Fig. 1). Consistent with the observation for BMI, the association of the GRS-34 and risk of overweight showed that each additional risk allele was associated with an 1.09-fold increased odds of overweight (95% CI: 1.04–1.16; *p* = 0.001).

## Discussion

Our findings regarding the association of established adult BMI associated loci with BMI and/or risk of overweight in adolescents reflect previous reports either on single pediatric cohorts (den Hoed et al., 2010; Bradfield et al*.,* 2012; Zhao et al., 2011) or large consortial meta-analyses (Bradfield et al., 2012). In the EYHS study, most of the BMI associated loci identified by GWAS in adults were also associated with anthropometric traits in children (den Hoed et al., 2010). In a case-control study of European American children, Zhao et al. (Zhao et al., 2011) reported evidence for association with common childhood obesity for nine of the 32 GIANT loci, while 28 of the 32 loci yielded consistent directional effects. The EGG consortium meta-analysis (Bradfield et al., 2012) also replicated robust associations for seven established adult BMI loci (*FTO, TMEM18, POMC, MC4R, FAIM2, TNNI3K* and *SEC16B*) with risk of childhood obesity; and identified two novel childhood obesity loci both of which had yielded directionally consistent effects in the GIANT meta-analysis of adult BMI (Speliotes et al., 2010). Consistency of directionality for the great majority of tested variants indicates that a large genetic component of BMI and obesity overlaps in children and adults (Bradfield et al., 2012).

Our study has a small sample size compared to both consortial meta-analyses, and power to confirm associations even at nominal significance level for all tested loci is relatively low. The effect sizes at the GIANT-identified loci require sample sizes that range from a few thousands to a few hundred thousand in order to achieve >80% power to detect association.

Despite the small effect sizes of established adult BMI associated variants, it has been shown that they have cumulative effects on BMI and obesity risk (Li et al., 2010). In our study, the combined effects of the 34 adult BMI and childhood obesity variants reported by the GIANT and EGG meta-analyses respectively were significantly associated with both BMI and risk of overweight, and the effect sizes are comparable to those reported in large meta-analyses of adults (Speliotes et al., 2010).

In this report, we investigated the individual and cumulative effects of established adult BMI and childhood obesity associated loci with BMI and overweight risk in a sample of Greek adolescents. We report evidence of nominal association for several loci and show that cumulatively tested variants are associated with both BMI and overweight risk. Our findings also support evidence for a large shared genetic component between adult and childhood BMI and obesity and validate the TEENAGE study as a cohort in which to study the genetics of anthropometric traits.

## Supporting Information

**Table S1** Descriptive characteristics of all subjects from the TEENAGE study.

**Table S2** TEENAGE results for BMI and childhood obesity associated loci with risk of obesity.

**Table S3a** TEENAGE association summary statistics for BMI and childhood obesity associated loci in males.

**Table S3b** TEENAGE association summary statistics for BMI and childhood obesity associated loci in females.

As a service to our authors and readers, this journal provides supporting information supplied by the authors. Such materials are peer-reviewed and may be re-organised for online delivery, but are not copy-edited or typeset. Technical support issues arising from supporting information (other than missing files) should be addressed to the authors.
