# Supplementary material for: Replication of Established Common Genetic Variants for Adult BMI and Childhood Obesity in Greek Adolescents: The TEENAGE Study
Source: Ann Hum Genet. 2013 Jan 24;77(3):268–74. doi: 10.1111/ahg.12012 (PMC3652032; doi:10.1111/ahg.12012)
Supplement: Supplementary file 1 [file ahg0077-0268-SD1.doc]

**Supplementary tables and figures**

**Supplementary Table 1** Descriptive characteristics of all subjects from the TEENAGE study

|  | All subjects | Males | Females |
| --- | --- | --- | --- |
| N (%) | 857 | 387 (45.2%) | 470 (54.8%) |
| Age (years) | 13.4 ± 0.9* | 13.4 ± 0.9 | 13.5 ± 0.9 |
| BMI (kg/m^2^) | 21.3 ± 3.6 | 21.5 ± 3.8 | 21.1 ± 3.4 |
| BMI categories |  |  |  |
| Normal weight (%) | 69.2 | 64.9 | 72.8 |
| Overweight (%) | 24.3 | 26.1 | 22.8 |
| Obese (%) | 6.5 | 9.0 | 4.5 |

* Mean ± standard deviation (sd)

**Supplementary Table 2** TEENAGE results for BMI and childhood obesity associated loci with risk of obesity.

| SNP | Nearest gene | Chr | Position (bp) | Alleles | | Obesity risk^a^ | | |
| --- | --- | --- | --- | --- | --- | --- | --- | --- |
|  |  |  |  | Effect | Other | OR | 95% CI | *p* |
| *GIANT (Speliotes et al., 2010)* ^e^ | | | | | | | | |
| rs1558902 | *FTO* | 16 | 52361075 | a | t | 1.30 | 0.86-1.96 | 0.248 |
| rs2867125 | *TMEM18* | 2 | 612827 | c | t | 1.17 | 0.69-1.97 | 0.528 |
| rs571312 | *MC4R* | 18 | 55990749 | a | c | 1.03 | 0.64-1.64 | 0.892 |
| rs10938397 | *GNPDA2* | 4 | 44877284 | g | a | 1.19 | 0.79-1.80 | 0.507 |
| **rs10767664** | ***BDNF*** | **11** | **27682562** | **a** | **t** | **1.72** | **1.00-2.95** | **0.028** |
| rs2815752 | *NEGR1* | 1 | 72585028 | a | g | 1.09 | 0.68-1.75 | 0.582 |
| rs7359397 | *SH2B1* | 16 | 28793160 | t | c | 0.96 | 0.60-1.51 | 0.856 |
| rs9816226 | *ETV5* | 3 | 187317193 | t | a | 0.93 | 0.55-1.58 | 0.778 |
| rs3817334 | *MTCH2* | 11 | 47607569 | t | c | 0.98 | 0.65-1.49 | 0.880 |
| rs29941 | *KCTD15* | 19 | 39001372 | g | a | 0.96 | 0.62-1.47 | 0.879 |
| rs543874 | *SEC16B* | 1 | 176156103 | g | a | 0.69 | 0.34-1.40 | 0.251 |
| rs987237 | *TFAP2B* | 6 | 50911009 | g | a | 1.61 | 0.99-2.61 | 0.102 |
| **rs7138803** | ***FAIM2*** | **12** | **48533735** | **a** | **g** | **1.58** | **1.05-2.40** | **0.025** |
| rs10150332 | *NRXN3* | 14 | 79006717 | c | t | 1.18 | 0.70-1.99 | 0.409 |
| rs713586 | *RBJ* | 2 | 25011512 | c | t | 1.45 | 0.96-2.19 | 0.095 |
| rs12444979 | *GPRC5BC* | 16 | 19841101 | c | t | 1.09 | 0.58-2.05 | 0.835 |
| rs2241423 | *MAP2K5* | 15 | 65873892 | g | a | 0.79 | 0.50-1.24 | 0.237 |
| rs2287019 | *QPCTL* | 19 | 50894012 | c | t | 1.46 | 0.79-2.67 | 0.227 |
| rs1514175 | *TNNI3K* | 1 | 74764232 | a | g | 1.02 | 0.67-1.55 | 0.982 |
| rs13107325 | *SLC39A8* | 4 | 103407732 | t | c | 1.07 | 0.54-2.13 | 0.817 |
| rs2112347 | *FLJ35779* | 5 | 75050998 | t | g | 0.98 | 0.65-1.49 | 0.994 |
| rs10968576 | *LRRN6C* | 9 | 28404339 | g | a | 0.71 | 0.41-1.22 | 0.299 |
| rs3810291 | *TMEM160* | 19 | 52260843 | a | g | 0.69 | 0.45-1.05 | 0.082 |
| rs887912 | *FANCL* | 2 | 59156381 | t | c | 0.89 | 0.55-1.42 | 0.558 |
| rs13078807 | *CADM2* | 3 | 85966840 | g | a | 1.21 | 0.75-1.96 | 0.565 |
| rs11847697 | *PRKD1* | 14 | 29584863 | t | c | 1.23 | 0.57-2.66 | 0.577 |
| rs2890652 | *LRP1B* | 2 | 142676401 | c | t | 1.02 | 0.58-1.80 | 0.916 |
| rs1555543 | *PTBP2* | 1 | 96717385 | c | a | 0.98 | 0.65-1.48 | 0.871 |
| rs4771122 | *MTIF3* | 13 | 26918180 | g | a | 0.86 | 0.51-1.46 | 0.565 |
| rs4836133 | *ZNF608* | 5 | 124360002 | a | c | 1.28 | 0.85-1.95 | 0.189 |
| rs4929949 | *RPL27A* | 11 | 8561169 | c | t | 0.99 | 0.65-1.52 | 0.963 |
| rs206936 | *NUDT3* | 6 | 34410847 | g | a | 0.77 | 0.46-1.28 | 0.360 |
| *EGG (Bradfield et al., 2012)* ^f^ | | | | | | | | |
| rs9568856 | *OLFM4* | 13 | 52962982 | A | G | 0.89 | 0.46-1.71 | 0.785 |
| rs9299 | *HOXB5* | 17 | 44024429 | T | C | 1.07 | 0.69-1.65 | 0.858 |

Chr, chromosome; bp, base pairs; EAF, effect allele frequency; SE, standard error; OR, odds ratio; CI; confidence interval.

Results were obtained using logistic regression analysis assuming an additive effect while controlling for age and sex. Allelic test *p*, OR and 95% CIs are shown for each single SNP. ORs are reported for the effect allele. Bold high-lighted loci yielded at least nominal evidence for association with obesity risk.

^a^ ‘Obesity risk*’* refers to the binary trait analysis: normal weight subjects vs. obese subjects.

**Supplementary Table 3a** TEENAGE association summary statistics for BMI and childhood obesity associated loci in males.

| SNP | Nearest gene | Chr | Position  (bp) | Alleles | | EAF | BMI^a^ | | | | | Overweight risk^c^ | | | Obesity risk^d^ | | |
| --- | --- | --- | --- | --- | --- | --- | --- | --- | --- | --- | --- | --- | --- | --- | --- | --- | --- |
|  |  |  |  | Effect | Other |  | beta^b^ | | SE^b^ | | *p* | OR | 95% CI | p | OR | 95% CI | p |
| *GIANT (Speliotes et al., 2010)* | | | | | | | | | | | | | | | | | |
| **rs1558902** | ***FTO*** | **16** | **52361075** | **a** | **t** | **0.51** | **0.62** | **0.31** | | **0.032** | | **1.45** | **1.04-2.02** | **0.028** | 1.09 | 0.64-1.87 | 0.718 |
| **rs2867125** | ***TMEM18*** | **2** | **612827** | **c** | **t** | 0.80 | 0.13 | 0.38 | | 0.571 | | 1.34 | 0.87-2.05 | 0.189 | 1.15 | 0.59-2.26 | 0.677 |
| rs571312 | *MC4R* | 18 | 55990749 | a | c | 0.25 | -0.03 | 0.36 | | 0.839 | | 1.02 | 0.70-1.49 | 0.928 | 0.87 | 0.46-1.64 | 0.633 |
| rs10938397 | *GNPDA2* | 4 | 44877284 | g | a | 0.43 | 0.07 | 0.31 | | 0.633 | | 0.94 | 0.68-1.32 | 0.727 | 1.07 | 0.62-1.83 | 0.832 |
| rs10767664 | *BDNF* | 11 | 27682562 | a | t | 0.75 | 0.67 | 0.36 | | 0.057 | | **1.58** | **1.06-2.36** | **0.022** | 1.77 | 0.89-3.52 | 0.092 |
| rs2815752 | *NEGR1* | 1 | 72585028 | a | g | 0.71 | -0.04 | 0.34 | | 0.934 | | 0.96 | 0.67-1.38 | 0.805 | 0.73 | 0.42-1.29 | 0.279 |
| rs7359397 | *SH2B1* | 16 | 28793160 | t | c | 0.29 | -0.51 | 0.35 | | 0.197 | | 0.88 | 0.61-1.27 | 0.527 | 0.73 | 0.39-1.36 | 0.337 |
| rs9816226 | *ETV5* | 3 | 187317193 | t | a | 0.82 | 0.22 | 0.41 | | 0.513 | | 0.99 | 0.64-1.53 | 0.982 | 1.11 | 0.54-2.29 | 0.748 |
| rs3817334 | *MTCH2* | 11 | 47607569 | t | c | 0.40 | 0.15 | 0.33 | | 0.513 | | 0.93 | 0.67-1.31 | 0.682 | 1.13 | 0.66-1.94 | 0.599 |
| rs29941 | *KCTD15* | 19 | 39001372 | g | a | 0.65 | -0.24 | 0.32 | | 0.458 | | 1.14 | 0.80-1.62 | 0.479 | 0.83 | 0.48-1.43 | 0.476 |
| rs543874 | *SEC16B* | 1 | 176156103 | g | a | 0.13 | 0.35 | 0.47 | | 0.413 | | 1.25 | 0.78-2.02 | 0.355 | 0.92 | 0.40-2.13 | 0.771 |
| rs987237 | *TFAP2B* | 6 | 50911009 | g | a | 0.21 | **1.22** | **0.39** | | **0.002** | | 1.44 | 0.97-2.15 | 0.066 | **1.96** | **1.08-3.55** | **0.026** |
| rs7138803 | *FAIM2* | 12 | 48533735 | a | g | 0.36 | **0.99** | **0.33** | | **0.003** | | **1.58** | **1.12-2.22** | **0.008** | **2.38** | **1.39-4.09** | **0.001** |
| rs10150332 | *NRXN3* | 14 | 79006717 | c | t | 0.16 | 0.31 | 0.41 | | 0.459 | | 1.36 | 0.88-2.10 | 0.161 | 1.33 | 0.67-2.65 | 0.410 |
| rs713586 | *RBJ* | 2 | 25011512 | c | t | 0.46 | 0.12 | 0.31 | | 0.727 | | 1.26 | 0.90-1.75 | 0.165 | 1.36 | 0.80-2.33 | 0.251 |
| rs12444979 | *GPRC5BC* | 16 | 19841101 | c | t | 0.88 | 0.56 | 0.49 | | 0.267 | | 1.46 | 0.84-2.51 | 0.171 | 1.69 | 0.65-4.42 | 0.269 |
| rs2241423 | *MAP2K5* | 15 | 65873892 | g | a | 0.77 | -0.02 | 0.35 | | 0.944 | | 1.09 | 0.73-1.62 | 0.702 | 0.87 | 0.47-1.61 | 0.628 |
| rs2287019 | *QPCTL* | 19 | 50894012 | c | t | 0.82 | 0.51 | 0.40 | | 0.188 | | 1.02 | 0.66-1.58 | 0.932 | 1.47 | 0.67-3.23 | 0.333 |
| rs1514175 | *TNNI3K* | 1 | 74764232 | a | g | 0.41 | 0.05 | 0.30 | | 0.792 | | 0.89 | 0.63-1.24 | 0.500 | 1.49 | 0.87-2.54 | 0.159 |
| rs13107325 | *SLC39A8* | 4 | 103407732 | t | c | 0.11 | 0.50 | 0.50 | | 0.284 | | 1.43 | 0.85-2.40 | 0.184 | 1.03 | 0.41-2.54 | 0.978 |
| rs2112347 | *FLJ35779* | 5 | 75050998 | t | g | 0.60 | -0.16 | 0.31 | | 0.786 | | 0.98 | 0.70-1.38 | 0.942 | 0.81 | 0.47-1.39 | 0.477 |
| rs10968576 | *LRRN6C* | 9 | 28404339 | g | a | 0.21 | 0.29 | 0.39 | | 0.331 | | 1.14 | 0.76-1.71 | 0.491 | 1.06 | 0.55-2.05 | 0.803 |
| rs3810291 | *TMEM160* | 19 | 52260843 | a | g | 0.68 | 0.17 | 0.33 | | 0.592 | | 1.05 | 0.74-1.50 | 0.775 | 0.94 | 0.54-1.66 | 0.847 |
| rs887912 | *FANCL* | 2 | 59156381 | t | c | 0.29 | -0.32 | 0.35 | | 0.409 | | 0.97 | 0.67-1.40 | 0.902 | 0.72 | 0.38-1.36 | 0.288 |
| rs13078807 | *CADM2* | 3 | 85966840 | g | a | 0.24 | 0.36 | 0.36 | | 0.307 | | 1.08 | 0.73-1.59 | 0.678 | 1.26 | 0.69-2.30 | 0.447 |
| rs11847697 | *PRKD1* | 14 | 29584863 | t | c | 0.06 | 0.71 | 0.66 | | 0.350 | | 1.14 | 0.58-2.27 | 0.707 | 1.14 | 0.38-3.41 | 0.834 |
| rs2890652 | *LRP1B* | 2 | 142676401 | c | t | 0.16 | 0.62 | 0.43 | | 0.139 | | 1.27 | 0.82-1.96 | 0.268 | 1.43 | 0.73-2.81 | 0.276 |
| rs1555543 | *PTBP2* | 1 | 96717385 | c | a | 0.49 | -0.05 | 0.31 | | 0.891 | | 1.03 | 0.74-1.44 | 0.881 | 0.97 | 0.57-1.65 | 0.860 |
| rs4771122 | *MTIF3* | 13 | 26918180 | g | a | 0.21 | 0.03 | 0.40 | | 0.906 | | 1.02 | 0.68-1.53 | 0.930 | 0.83 | 0.41-1.65 | 0.542 |
| rs4836133 | *ZNF608* | 5 | 124360002 | a | c | 0.53 | 0.46 | 0.31 | | 0.159 | | 1.22 | 0.88-1.70 | 0.232 | 1.53 | 0.88-2.64 | 0.110 |
| rs4929949 | *RPL27A* | 11 | 8561169 | c | t | 0.37 | -0.08 | 0.34 | | 0.883 | | 1.08 | 0.76-1.52 | 0.634 | 1.34 | 0.78-2.31 | 0.236 |
| rs206936 | *NUDT3* | 6 | 34410847 | g | a | 0.23 | 0.10 | 0.36 | | 0.719 | | 0.89 | 0.60-1.32 | 0.559 | 0.93 | 0.49-1.76 | 0.856 |
| *EGG (Bradfield et al., 2012)* | | | | | | | | | | | | | | | | | |
| rs9568856 | *OLFM4* | 13 | 52962982 | A | G | 0.12 | 0.41 | 0.46 | | 0.341 | | 1.15 | 0.70-1.90 | 0.602 | 0.83 | 0.34-2.03 | 0.662 |
| rs9299 | *HOXB5* | 17 | 44024429 | T | C | 0.68 | 0.15 | 0.33 | | 0.600 | | 1.12 | 0.79-1.60 | 0.531 | 1.21 | 0.67-2.17 | 0.541 |

Chr, chromosome; bp, base pairs; EAF, effect allele frequency; SE, standard error; OR, odds ratio; CI; confidence interval.

Results were obtained using linear regression and logistic regression analysis assuming an additive effect while controlling for age. Allelic test *p*, beta and SE, OR and 95% CIs are shown for each single SNP. Effect sizes (beta) and ORs are reported for the effect allele. Bold high-lighted loci yielded at least nominal evidence for association with BMI and/or overweight risk.

^a^ ‘BMI*’* refers to the linear regression analysis of each variant with BMI.

^b^ Effect sizes (beta) and SE are given for untransformed BMI (kg/m^2^).

^c^ ‘Overweight risk’ refers to the binary trait analysis: normal weight vs. overweight subjects (obese subjects were also classified as overweight).

^d^ ‘Obesity risk’ refers to the binary trait analysis: normal weight vs. obese subjects.

**Supplementary Table 3b** TEENAGE association summary statistics for BMI and childhood obesity associated loci in females.

| SNP | Nearest gene | Chr | Position  (bp) | Alleles | | EAF | BMI^a^ | | | | | Overweight risk^c^ | | | | Obesity risk^d^ | | | |  |
| --- | --- | --- | --- | --- | --- | --- | --- | --- | --- | --- | --- | --- | --- | --- | --- | --- | --- | --- | --- | --- |
|  |  |  |  | Effect | Other |  | beta^b^ | | SE^b^ | | *p* | OR | | 95% CI | p | OR | 95% CI | | p |  |
| *GIANT (Speliotes et al., 2010)* | | | | | | | | | | | | | | | | | | | |  |
| **rs1558902** | ***FTO*** | **16** | **52361075** | **a** | **t** | **0.45** | **0.53** | **0.23** | | **0.016** | | 1.21 | | 0.88-1.65 | 0.241 | 1.58 | 0.82-3.06 | | 0.188 |  |
| **rs2867125** | ***TMEM18*** | **2** | **612827** | **c** | **t** | **0.81** | **0.74** | **0.30** | | **0.014** | | **1.61** | | **1.06-2.47** | **0.022** | 1.20 | 0.52-2.80 | | 0.611 |  |
| rs571312 | *MC4R* | 18 | 55990749 | a | c | 0.26 | 0.10 | 0.27 | | 0.763 | | 0.99 | | 0.70-1.42 | 0.956 | 1.33 | 0.65-2.70 | | 0.392 |  |
| rs10938397 | *GNPDA2* | 4 | 44877284 | g | a | 0.41 | 0.13 | 0.25 | | 0.618 | | 1.02 | | 0.74-1.40 | 0.930 | 1.33 | 0.69-2.56 | | 0.413 |  |
| rs10767664 | *BDNF* | 11 | 27682562 | a | t | 0.75 | 0.0004 | 0.29 | | 0.947 | | 0.90 | | 0.63-1.27 | 0.533 | 1.77 | 0.72-4.32 | | 0.160 |  |
| rs2815752 | *NEGR1* | 1 | 72585028 | a | g | 0.73 | 0.12 | 0.27 | | 0.789 | | 0.93 | | 0.66-1.32 | 0.807 | **3.15** | **1.10-9.02** | | **0.018** |  |
| rs7359397 | *SH2B1* | 16 | 28793160 | t | c | 0.28 | 0.25 | 0.28 | | 0.339 | | 1.07 | | 0.76-1.51 | 0.790 | 1.34 | 0.67-2.69 | | 0.432 |  |
| rs9816226 | *ETV5* | 3 | 187317193 | t | a | 0.82 | 0.0005 | 0.33 | | 0.899 | | 1.07 | | 0.71-1.62 | 0.806 | 0.72 | 0.33-1.56 | | 0.340 |  |
| rs3817334 | *MTCH2* | 11 | 47607569 | t | c | 0.42 | -0.07 | 0.24 | | 0.919 | | **0.72** | | **0.53-1.00** | **0.049** | 0.84 | 0.43-1.64 | | 0.679 |  |
| rs29941 | *KCTD15* | 19 | 39001372 | g | a | 0.69 | 0.29 | 0.27 | | 0.315 | | 1.28 | | 0.91-1.81 | 0.210 | 1.36 | 0.65-2.86 | | 0.534 |  |
| rs543874 | *SEC16B* | 1 | 176156103 | g | a | 0.12 | 0.03 | 0.37 | | 0.759 | | 0.78 | | 0.47-1.28 | 0.371 | 0.38 | 0.09-1.59 | | 0.179 |  |
| rs987237 | *TFAP2B* | 6 | 50911009 | g | a | 0.16 | -0.21 | 0.33 | | 0.466 | | 1.03 | | 0.68-1.56 | 0.987 | 0.97 | 0.39-2.38 | | 0.839 |  |
| rs7138803 | *FAIM2* | 12 | 48533735 | a | g | 0.38 | 0.15 | 0.25 | | 0.544 | | 1.10 | | 0.80-1.51 | 0.507 | 0.89 | 0.45-1.78 | | 0.791 |  |
| rs10150332 | *NRXN3* | 14 | 79006717 | c | t | 0.18 | -0.16 | 0.31 | | 0.553 | | 1.01 | | 0.68-1.51 | 0.941 | 1.11 | 0.49-2.53 | | 0.737 |  |
| rs713586 | *RBJ* | 2 | 25011512 | c | t | 0.42 | 0.37 | 0.24 | | 0.137 | | **1.35** | | **0.99-1.85** | **0.049** | 1.48 | 0.77-2.86 | | 0.199 |  |
| rs12444979 | *GPRC5BC* | 16 | 19841101 | c | t | 0.87 | 0.06 | 0.35 | | 0.880 | | 1.03 | | 0.64-1.64 | 0.852 | 0.66 | 0.28-1.54 | | 0.381 |  |
| rs2241423 | *MAP2K5* | 15 | 65873892 | g | a | 0.75 | 0.15 | 0.30 | | 0.565 | | 1.09 | | 0.76-1.58 | 0.506 | 0.64 | 0.32-1.29 | | 0.233 |  |
| rs2287019 | *QPCTL* | 19 | 50894012 | c | t | 0.84 | 0.57 | 0.32 | | 0.066 | | 1.50 | | 0.96-2.36 | 0.077 | 1.43 | 0.55-3.76 | | 0.483 |  |
| rs1514175 | *TNNI3K* | 1 | 74764232 | a | g | 0.40 | 0.01 | 0.24 | | 0.818 | | 1.04 | | 0.76-1.43 | 0.694 | 0.47 | 0.22-1.02 | | 0.071 |  |
| rs13107325 | *SLC39A8* | 4 | 103407732 | t | c | 0.10 | 0.55 | 0.41 | | 0.160 | | 1.23 | | 0.74-2.03 | 0.348 | 1.14 | 0.39-3.33 | | 0.635 |  |
| rs2112347 | *FLJ35779* | 5 | 75050998 | t | g | 0.60 | 0.19 | 0.24 | | 0.436 | | 1.04 | | 0.76-1.42 | 0.814 | 1.34 | 0.67-2.69 | | 0.348 |  |
| **rs10968576** | ***LRRN6C*** | **9** | **28404339** | **g** | **a** | **0.23** | **-0.68** | **0.29** | | **0.019** | | 0.83 | | 0.57-1.21 | 0.298 | **0.37** | **0.13-1.07** | | **0.048** |  |
| rs3810291 | *TMEM160* | 19 | 52260843 | a | g | 0.68 | -0.31 | 0.26 | | 0.336 | | 0.80 | | 0.58-1.12 | 0.155 | **0.44** | **0.23-0.86** | | **0.008** |  |
| rs887912 | *FANCL* | 2 | 59156381 | t | c | 0.28 | -0.04 | 0.26 | | 0.916 | | 0.95 | | 0.67-1.35 | 0.657 | 1.18 | 0.58-2.40 | | 0.731 |  |
| rs13078807 | *CADM2* | 3 | 85966840 | g | a | 0.21 | 0.13 | 0.29 | | 0.645 | | 1.11 | | 0.76-1.61 | 0.731 | 1.04 | 0.46-2.33 | | 0.865 |  |
| rs11847697 | *PRKD1* | 14 | 29584863 | t | c | 0.07 | 0.36 | 0.48 | | 0.446 | | 0.92 | | 0.49-1.70 | 0.645 | 1.50 | 0.50-4.48 | | 0.581 |  |
| rs2890652 | *LRP1B* | 2 | 142676401 | c | t | 0.15 | -0.31 | 0.35 | | 0.399 | | 0.86 | | 0.55-1.34 | 0.574 | 0.47 | 0.14-1.56 | | 0.209 |  |
| rs1555543 | *PTBP2* | 1 | 96717385 | c | a | 0.51 | 0.40 | 0.24 | | 0.063 | | 1.28 | | 0.94-1.75 | 0.109 | 1.02 | 0.53-1.96 | | 0.991 |  |
| rs4771122 | *MTIF3* | 13 | 26918180 | g | a | 0.20 | -0.20 | 0.31 | | 0.450 | | 0.87 | | 0.59-1.29 | 0.544 | 0.91 | 0.40-2.10 | | 0.894 |  |
| rs4836133 | *ZNF608* | 5 | 124360002 | a | c | 0.54 | 0.32 | 0.25 | | 0.166 | | 1.09 | | 0.80-1.49 | 0.566 | 1.01 | 0.52-1.95 | | 0.914 |  |
| rs4929949 | *RPL27A* | 11 | 8561169 | c | t | 0.38 | -0.42 | 0.26 | | 0.138 | | 0.88 | | 0.64-1.22 | 0.379 | 0.62 | 0.30-1.29 | | 0.141 |  |
| rs206936 | *NUDT3* | 6 | 34410847 | g | a | 0.24 | -0.11 | 0.29 | | 0.828 | | 0.85 | | 0.58-1.22 | 0.373 | 0.56 | 0.23-1.37 | | 0.189 |  |
| *EGG (Bradfield et al., 2012)* | | | | | | | | | | | | | | | | | | | |  |
| rs9568856 | *OLFM4* | 13 | 52962982 | A | G | 0.13 | 0.61 | 0.37 | | 0.100 | | 1.11 | 0.71-1.75 | | 0.483 | 1.04 | | 0.39-2.75 | 0.847 | |
| rs9299 | *HOXB5* | 17 | 44024429 | T | C | 0.35 | 0.18 | 0.25 | | 0.436 | | 1.03 | 0.75-1.43 | | 0.764 | 0.84 | | 0.43-1.65 | 0.672 | |

Chr, chromosome; bp, base pairs; EAF, effect allele frequency; SE, standard error; OR, odds ratio; CI; confidence interval.

Results were obtained using linear regression and logistic regression analysis assuming an additive effect while controlling for age. Allelic test *p*, beta and SE, OR and 95% CIs are shown for each single SNP. Effect sizes (beta) and ORs are reported for the effect allele. Bold high-lighted loci yielded at least nominal evidence for association with BMI and/or overweight risk.

^a^ ‘BMI*’* refers to the linear regression analysis of each variant with BMI.

^b^ Effect sizes (beta) and SE are given for untransformed BMI (kg/m^2^).

^c^ ‘Overweight risk’ refers to the binary trait analysis: normal weight vs. overweight subjects (obese subjects were also classified as overweight).

^d^ ‘Obesity risk’ refers to the binary trait analysis: normal weight vs. obese subjects.
